# Supplementary material for: Proteo-Transcriptomic Analysis of the Venom Gland of the Cone Snail Cylinder canonicus Reveals the Origin of the Predatory-Evoked Venom
Source: Toxins (Basel). 2025 Mar 2;17(3):119. doi: 10.3390/toxins17030119 (PMC11946857; doi:10.3390/toxins17030119)
Supplement: Supplementary file 1 [file toxins-17-00119-s001.zip › toxins-3458638-supplementary/toxins-3458638-supplementary file S1.docx]

Supplementary Materials: Proteo-Transcriptomic Analysis of the Venom Gland of the Cone Snail *Cylinder canonicus* Reveals the Origin of the Predatory-Evoked Venom

Zahrmina Ratibou, Anicet E. T. Ebou, Claudia Muracciole Bich, Fabrice Saintmont, Gilles Valette, Guillaume Cazals, Dominique K. Koua, Nicolas Inguimbert, Sébastien Dutertre

Supplemental file S1 - Sequence alignment of 108 conotoxin precursors through transcriptomics (Figures S1-S22) and proteomics approach (Figures S23-S27).

Content

[Figure S1. Sequence alignment of four conotoxin precursors of the A gene superfamily (Can01-Can04). 3](#_Toc187825241)

[Figure S2. Sequence alignment of two conotoxin precursors the B1 gene superfamily (Can05-Can06). 3](#_Toc187825242)

[Figure S3. Conotoxin precursor belonging to the D gene superfamily (Can07). 3](#_Toc187825243)

[Figure S4. Conotoxin precursor belonging of the F gene superfamily (Can08). 3](#_Toc187825244)

[Figure S5. Sequence alignment of three conotoxin precursors of the H gene superfamily (Can09-Can11). 4](#_Toc187825245)

[Figure S6. Sequence alignment of two conotoxin precursors of the I2 gene superfamily (Can12-Can13). 4](#_Toc187825246)

[Figure S7. Sequence alignment of two conotoxin precursors of the I3 gene superfamily (Can14-Can15). 4](#_Toc187825247)

[Figure S8. Sequence alignment of 14 conotoxin precursors of the M gene superfamily (Can16-Can29). 4](#_Toc187825248)

[Figure S9. Sequence alignment of 12 conotoxin precursors of the O1 gene superfamily (Can30-Can41). 5](#_Toc187825249)

[Figure S10. Sequence alignment of 12 conotoxin precursors of the O2 gene superfamily (Can42-Can53). 5](#_Toc187825250)

[Figure S11. Sequence alignment of one conotoxin of the O3 gene superfamily (Can54). 6](#_Toc187825251)

[Figure S12. Sequence alignment of five conotoxin precursors of the P gene superfamily (Can55-Can59). 6](#_Toc187825252)

[Figure S13. Sequence alignment of two conotoxin precursors of the S gene superfamily (Can60-Can61). 6](#_Toc187825253)

[Figure S14. Sequence alignment of eight conotoxin precursors of the T gene superfamily (Can62-Can69). 6](#_Toc187825254)

[Figure S15. Sequence of a conotoxin precursor belonging to the U gene superfamily (Can70). 7](#_Toc187825255)

[Figure S16. Sequence alignment of seven conotoxin precursors of the Con-ikot-ikot gene superfamily (Can71-Can77). 7](#_Toc187825256)

[Figure S17. Sequence alignment of two conotoxin precursors of the Con-insulins gene superfamily (Can78-Can79). 7](#_Toc187825257)

[Figure S18. Sequence alignment of 12 conotoxin precursors of the Conkunitzin gene superfamily (Can80-91). 8](#_Toc187825258)

[Figure S19. Sequence alignment of three conotoxin precursors of the Elevenin gene superfamily (Can92-Can94). 8](#_Toc187825259)

[Figure S20. Sequence of a conotoxin precursor belonging to the Conopressin-conophysin gene superfamily (Can95). 8](#_Toc187825260)

[Figure S21. Sequence alignment of two conotoxin precursors of the Conorfamide gene superfamily. 9](#_Toc187825261)

[Figure S22. Sequence a conotoxin precursor (Can98) belonging to the neuropeptide prohormone 4 gene superfamily. Gene superfamily attribution was annotated by similarity with a neuropeptide prohormone previously identified in another gastropod, the golden apple snail *Pomacea* *canaliculate* (accession XM_025248922.1, Zhou et al., *Mitochondrial DNA Part A,* 2016). 9](#_Toc187825262)

[Figure S23. Sequence alignment of Can99, an E-conotoxin precursor identified in this study in the venom of *C. canonicus* with the E-conotoxin Vc1.1 from *C. victoriae* (Robinson et al., *PLOS ONE* 2014)*.* 9](#_Toc187825263)

[Figure S24. Sequence alignment of two I1-conotoxin precursors, identified in this study, in the venom of *C. canonicus* (Can100-Can101) with the I1-conotoxin Pmag02 from *C. ebraeus* (Pardos-Blas et al., *Mar. Drugs* 2022). 9](#_Toc187825264)

[Figure S25. Sequence alignment of four O1-conotoxin precursors, from in this study, in the venom of *C. canonicus* (Can102-Can105) with the O1-conotoxin ω-TeA61 from *C. textile* (Luo et al., *J. Pept. Sci.* 2006)*.* 10](#_Toc187825265)

[Figure S26. Sequence alignment of an O2-conotoxin precursor, from in this study, in the venom of *C. canonicus* (Can106) with the O2-conotoxin Vc6.7 from *C. victoriae* (Safavi-Hemani et al., *J. Proteome Res.* 2011)*.* 10](#_Toc187825266)

[Figure S27. Sequence alignment of four T-conotoxin precursors, from in this study, in the venom of *C. canonicus* (Can107-Can108) with the T-conotoxin Vc5.1 from *C. victoriae* (Jakubowski et al., *J. Mass Spectrom.* 2004). 10](#_Toc187825267)

# Transcriptomics

## A SUPERFAMILY


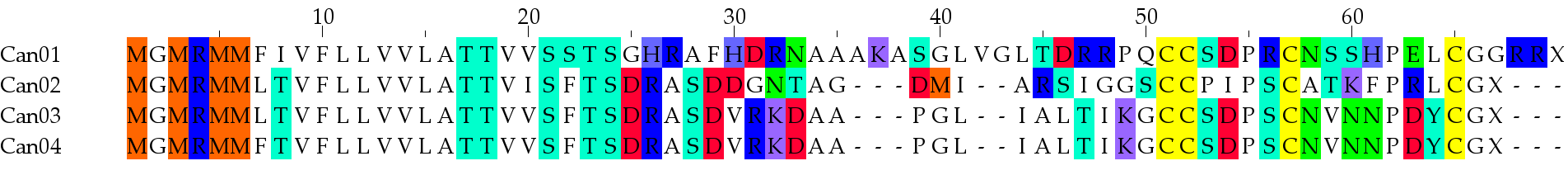


Figure S1. Sequence alignment of four conotoxin precursors of the A gene superfamily (Can01-Can04).

## B1 SUPERFAMILY


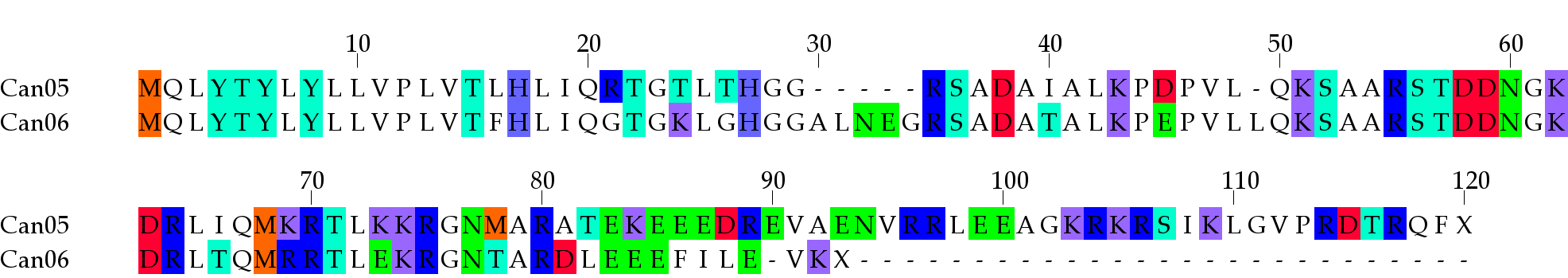


Figure S2. Sequence alignment of two conotoxin precursors the B1 gene superfamily (Can05-Can06).

## D SUPERFAMILY


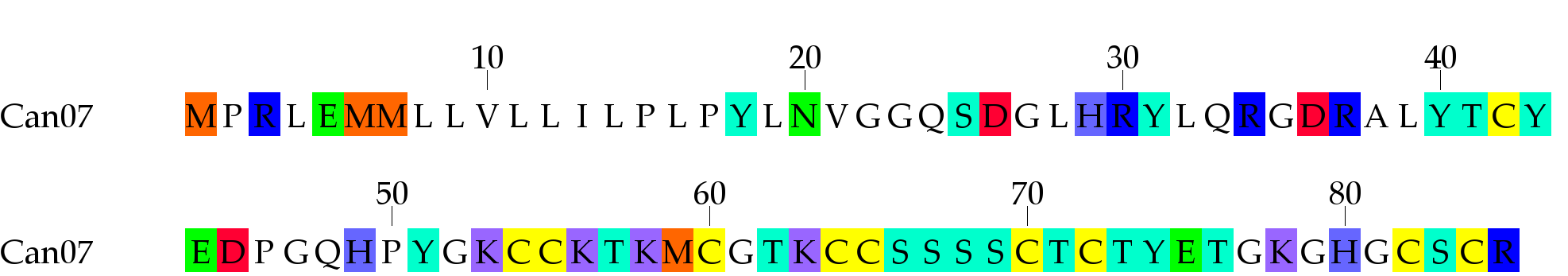


Figure S3. Conotoxin precursor belonging to the D gene superfamily (Can07).

## F SUPERFAMILY


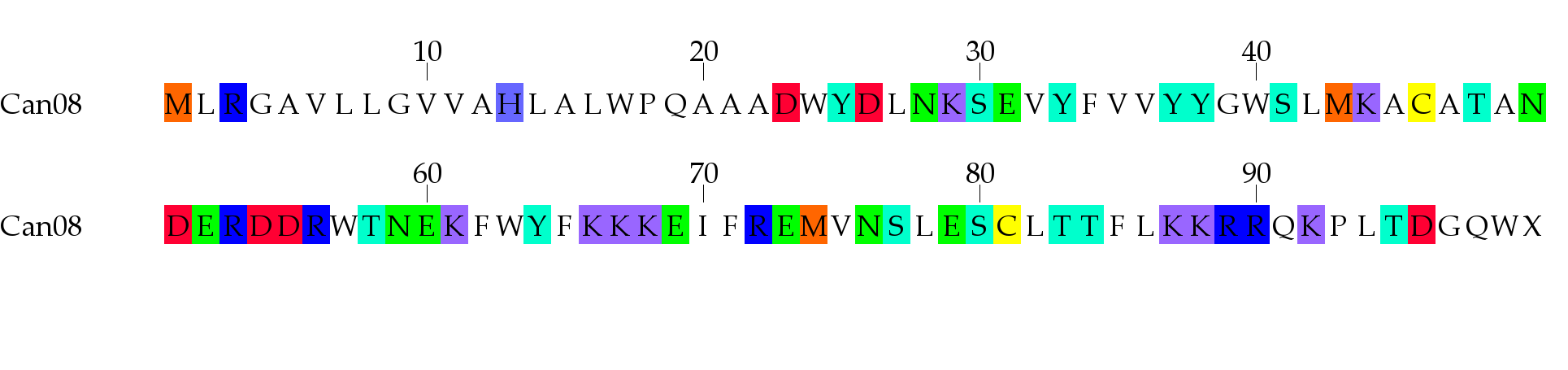


Figure S4. Conotoxin precursor belonging of the F gene superfamily (Can08).

## H SUPERFAMILY


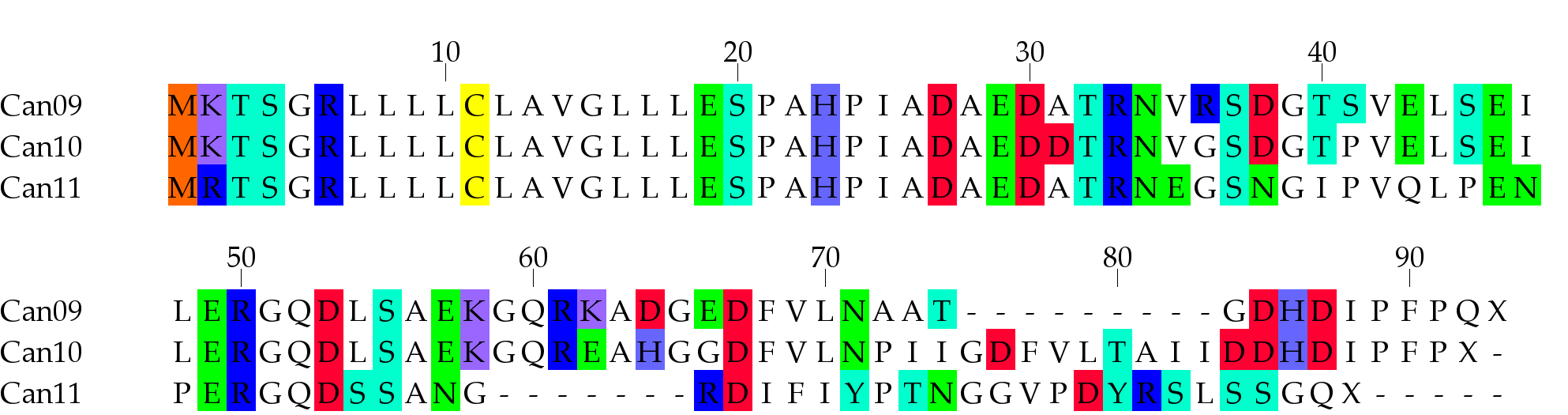


Figure S5. Sequence alignment of three conotoxin precursors of the H gene superfamily (Can09-Can11).

## I2 SUPERFAMILY


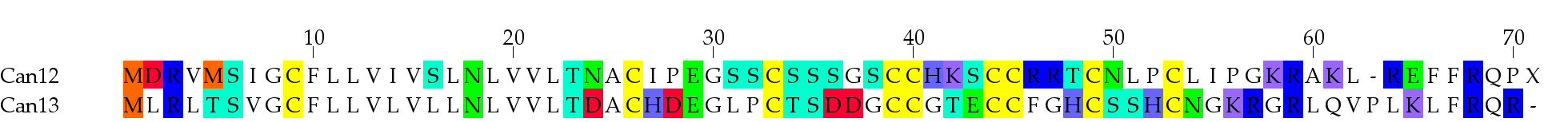


Figure S6. Sequence alignment of two conotoxin precursors of the I2 gene superfamily (Can12-Can13).

## I3 SUPERFAMILY


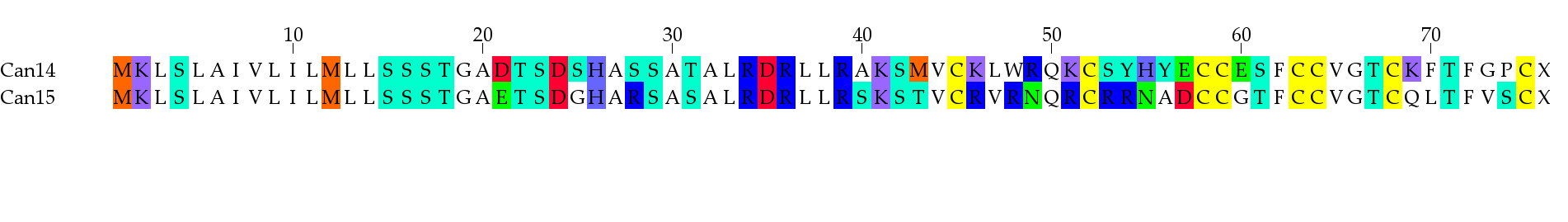


Figure S7. Sequence alignment of two conotoxin precursors of the I3 gene superfamily (Can14-Can15).

## M SUPERFAMILY


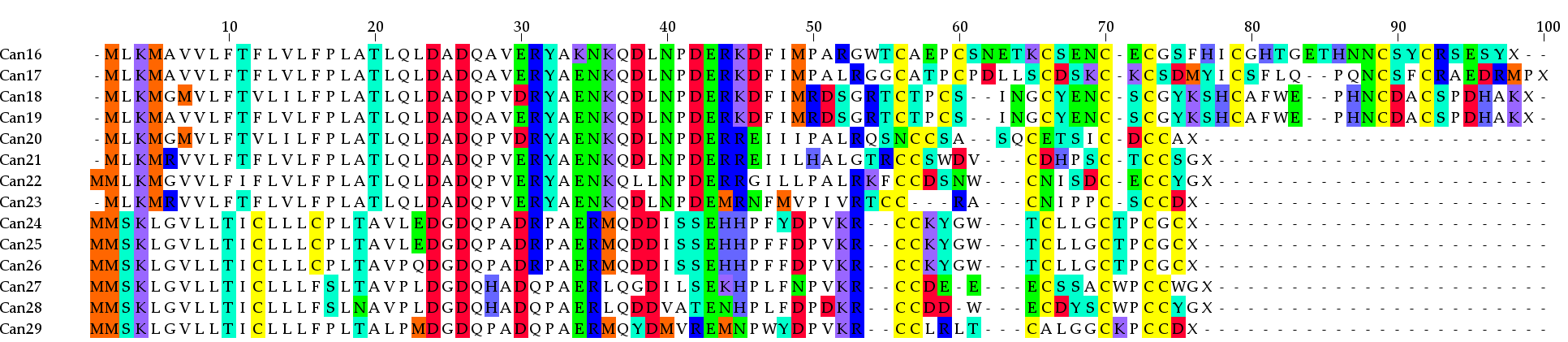


Figure S8. Sequence alignment of 14 conotoxin precursors of the M gene superfamily (Can16-Can29).

## O1 SUPERFAMILY


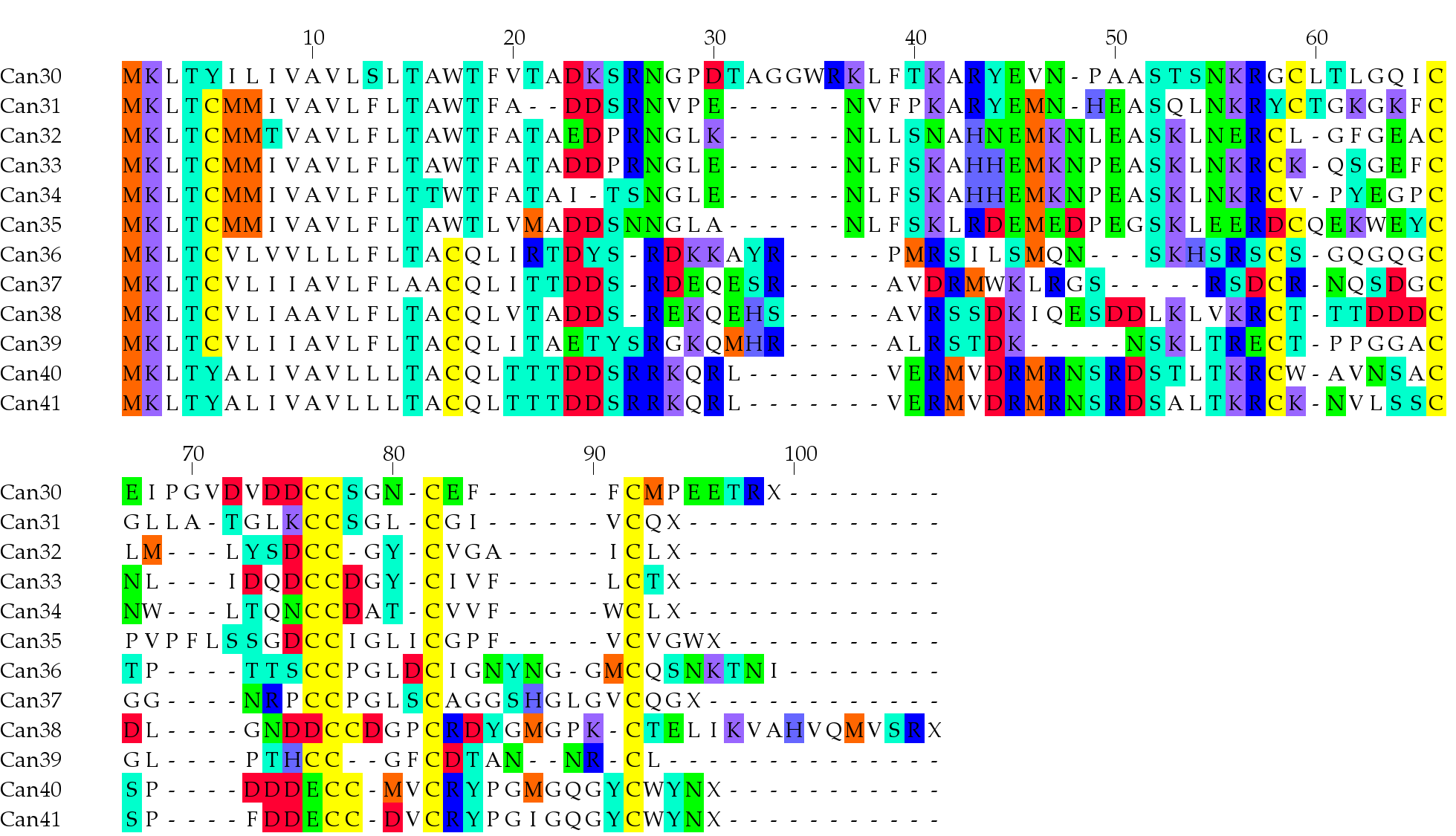


Figure S9. Sequence alignment of 12 conotoxin precursors of the O1 gene superfamily (Can30-Can41).

## O2 SUPERFAMILY


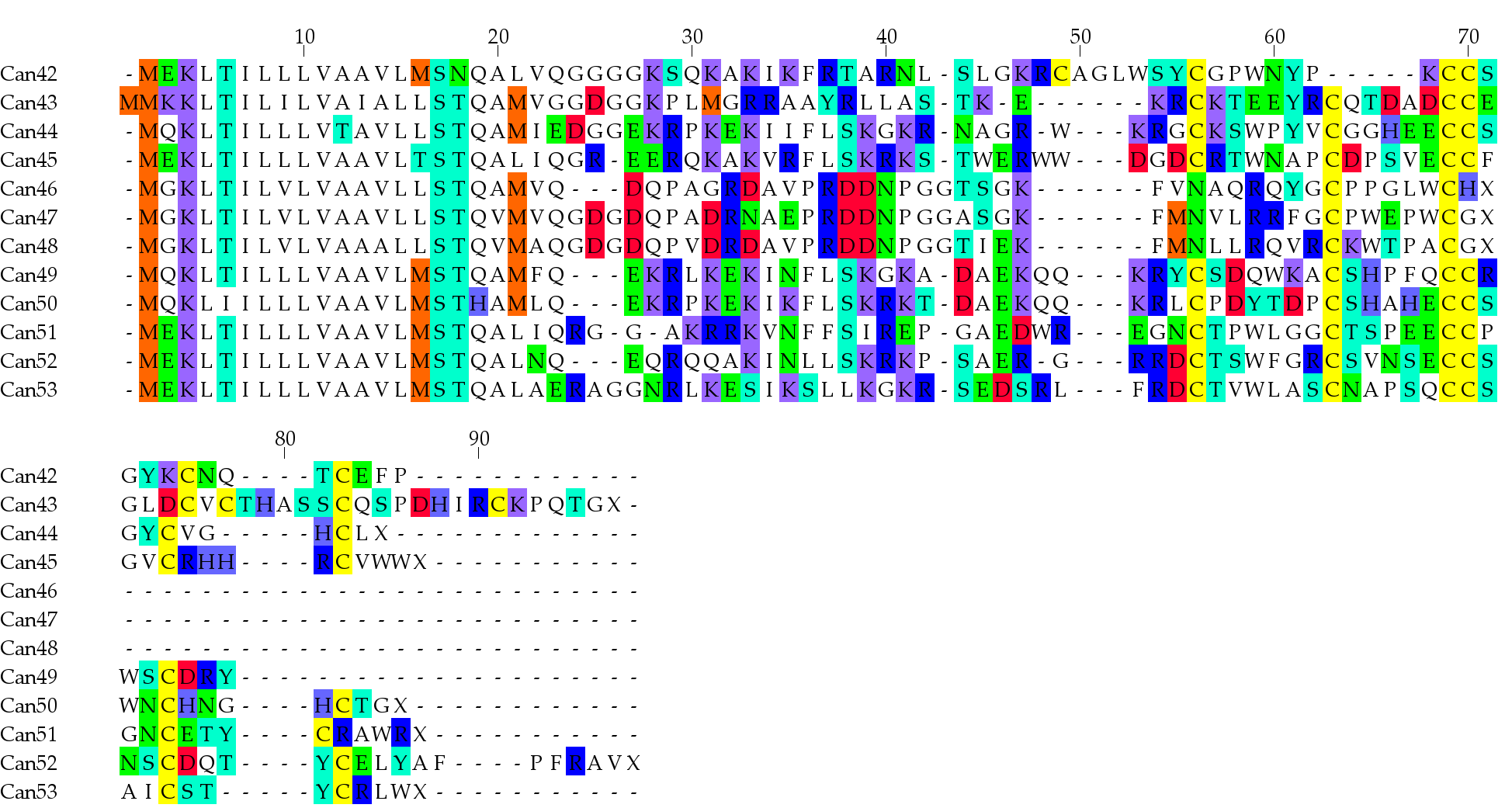


Figure S10. Sequence alignment of 12 conotoxin precursors of the O2 gene superfamily (Can42-Can53).

## O3 SUPERFAMILY


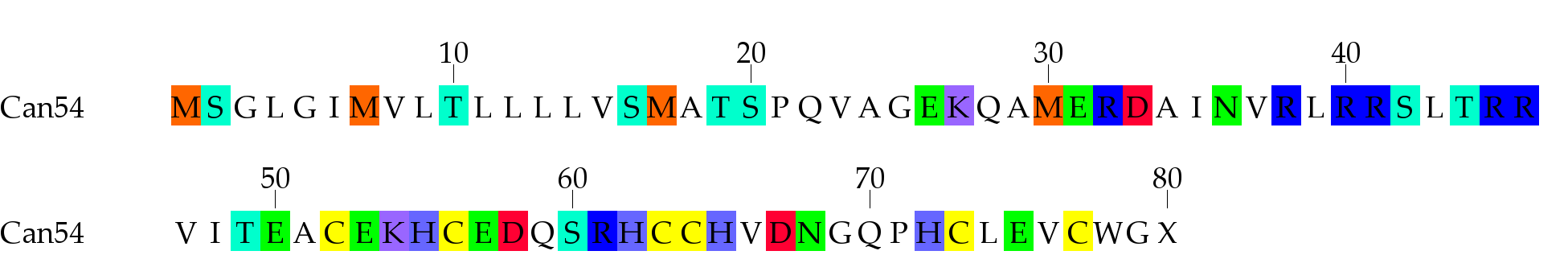


Figure S11. Sequence alignment of one conotoxin of the O3 gene superfamily (Can54).

## P SUPERFAMILY


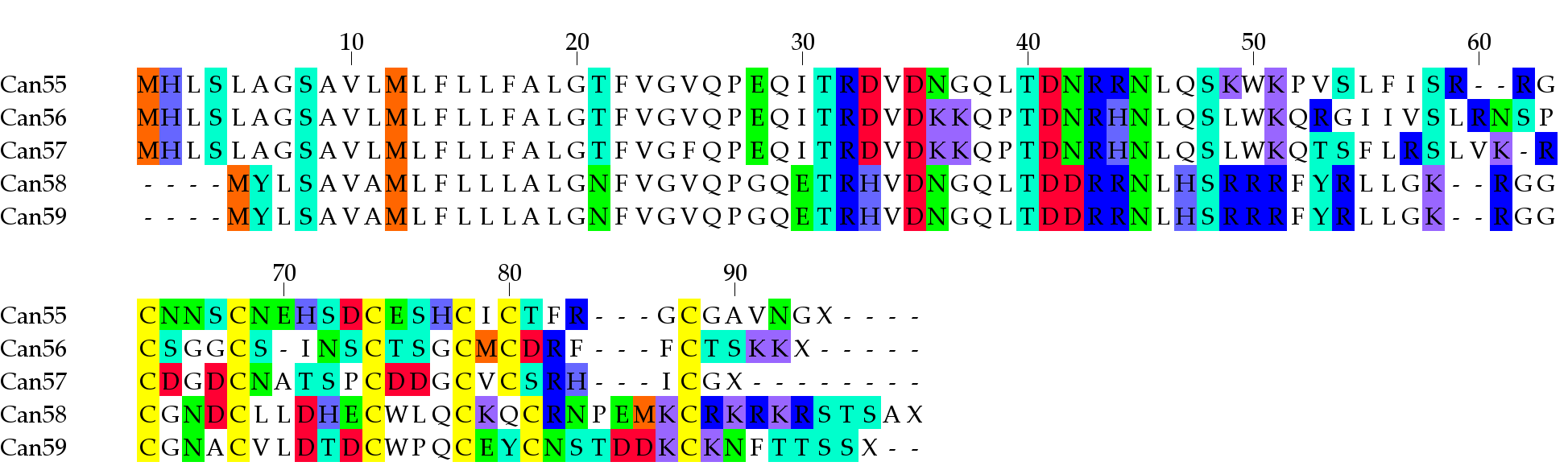


Figure S12. Sequence alignment of five conotoxin precursors of the P gene superfamily (Can55-Can59).

## S SUPERFAMILY


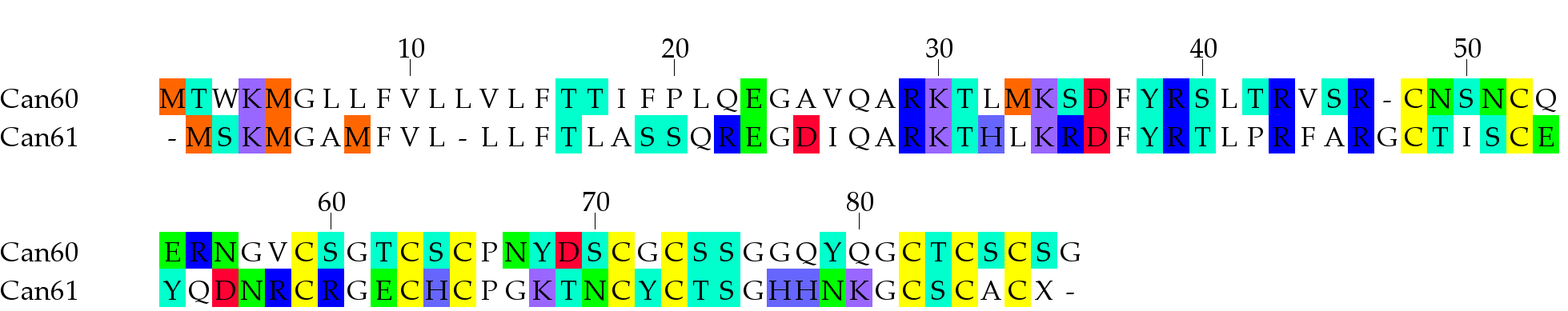


Figure S13. Sequence alignment of two conotoxin precursors of the S gene superfamily (Can60-Can61).

## T SUPERFAMILY


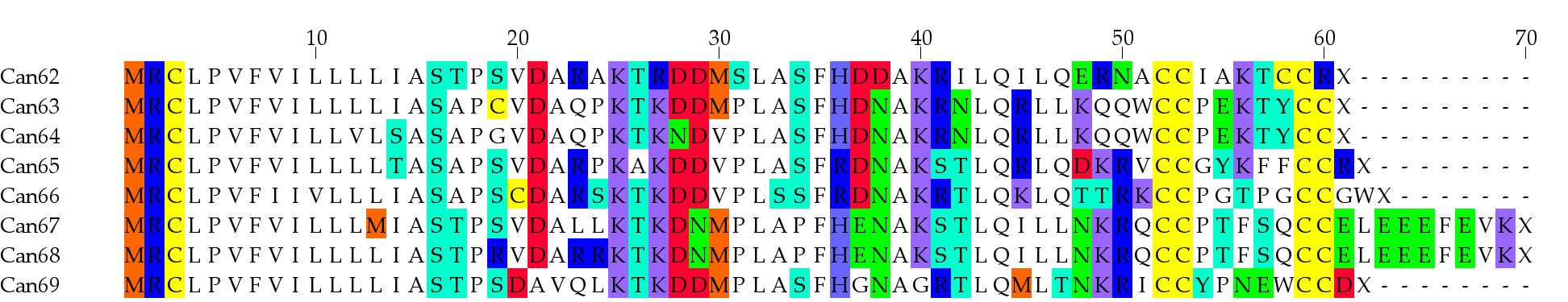


Figure S14. Sequence alignment of eight conotoxin precursors of the T gene superfamily (Can62-Can69).

## U SUPERFAMILY


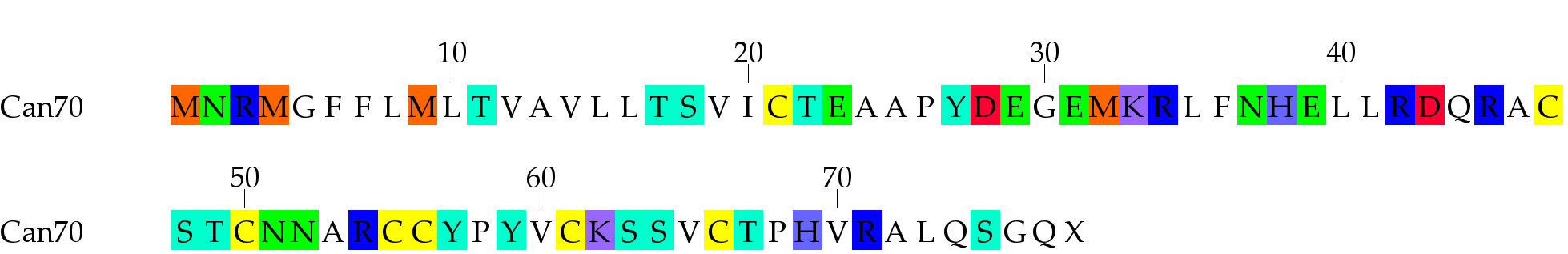


Figure S15. Sequence of a conotoxin precursor belonging to the U gene superfamily (Can70).

## Con-ikot-ikot SUPERFAMILY


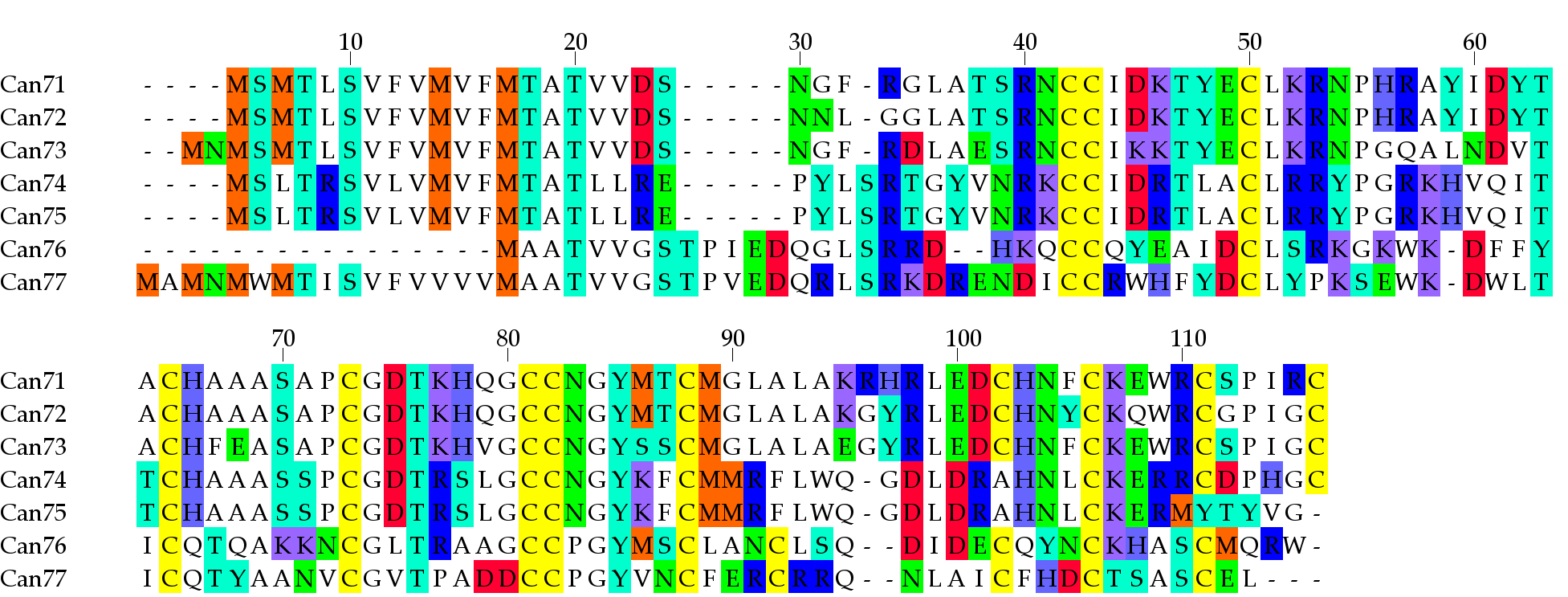


Figure S16. Sequence alignment of seven conotoxin precursors of the Con-ikot-ikot gene superfamily (Can71-Can77).

## Con-insulin SUPERFAMILY


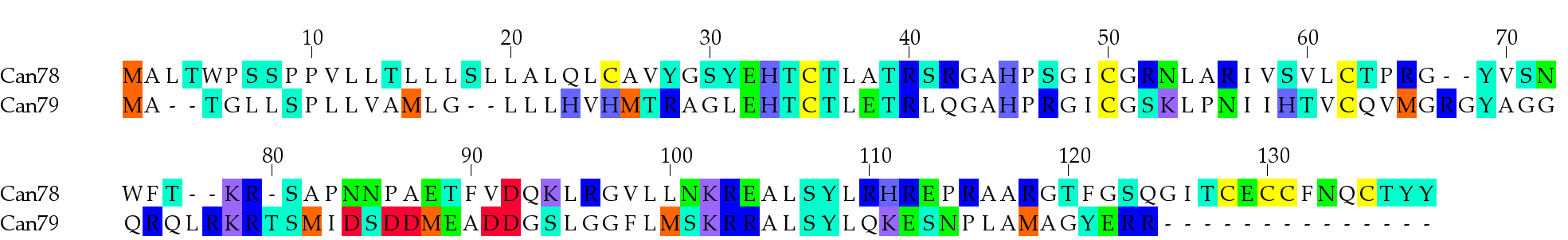


Figure S17. Sequence alignment of two conotoxin precursors of the Con-insulins gene superfamily (Can78-Can79).

## Conkunitzin SUPERFAMILY


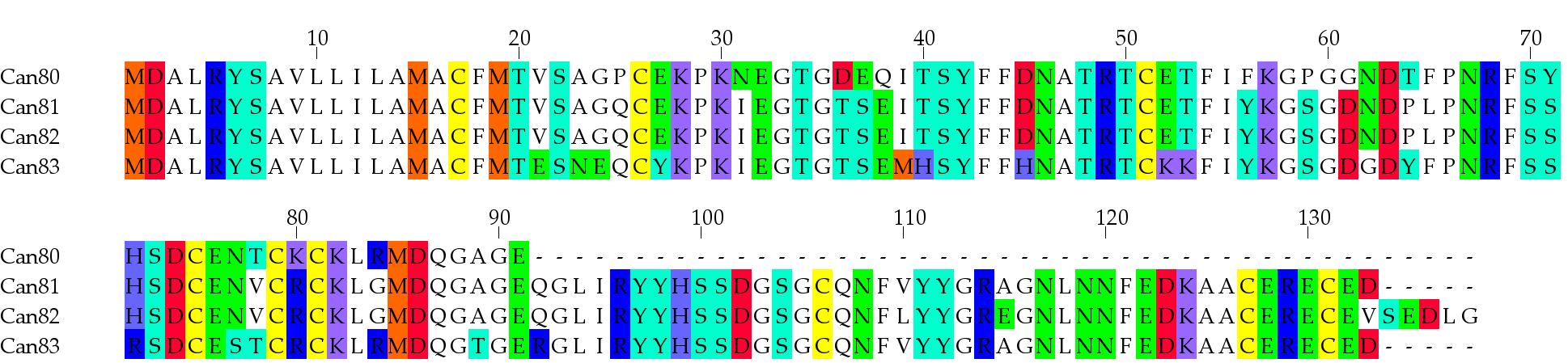


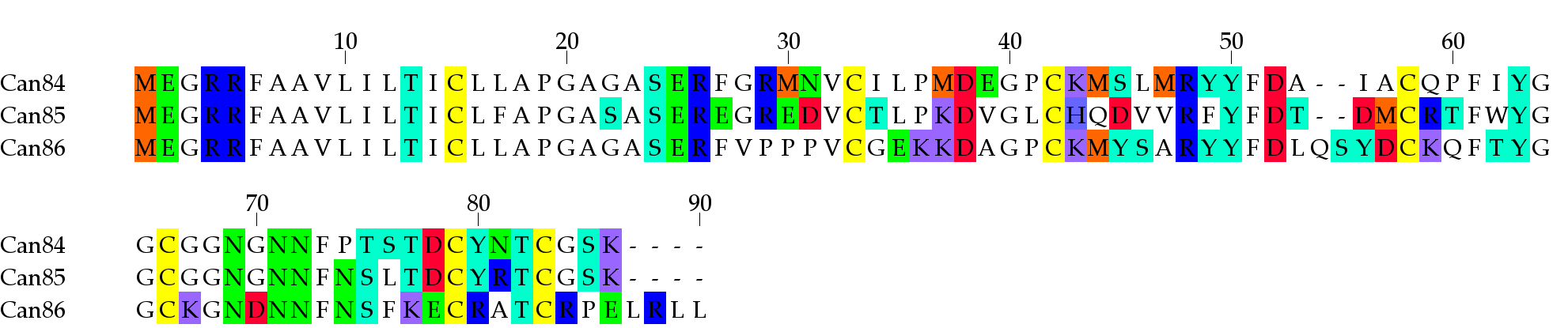

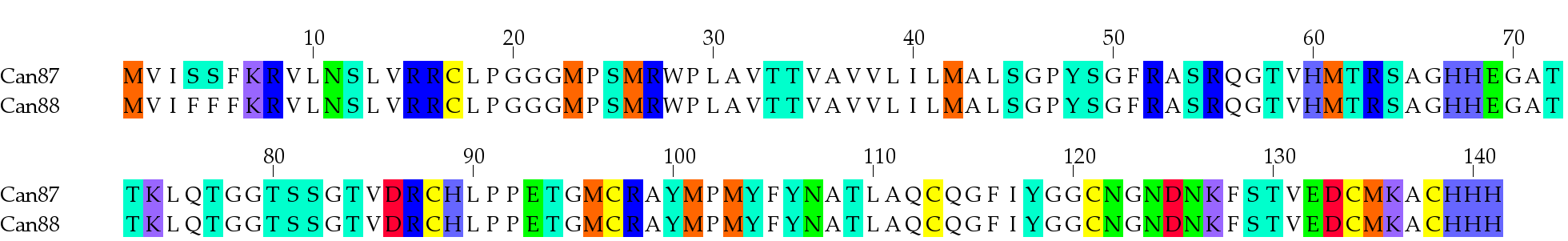

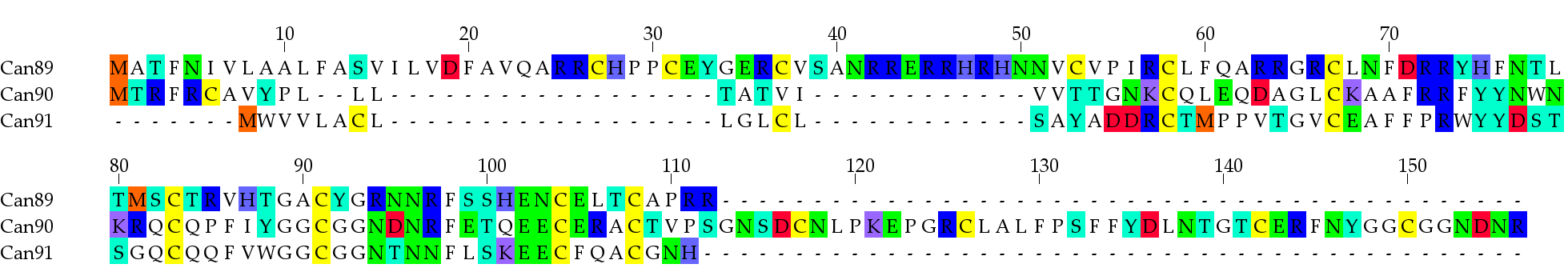


Figure S18. Sequence alignment of 12 conotoxin precursors of the Conkunitzin gene superfamily (Can80-91).

## Elevenin SUPERFAMILY


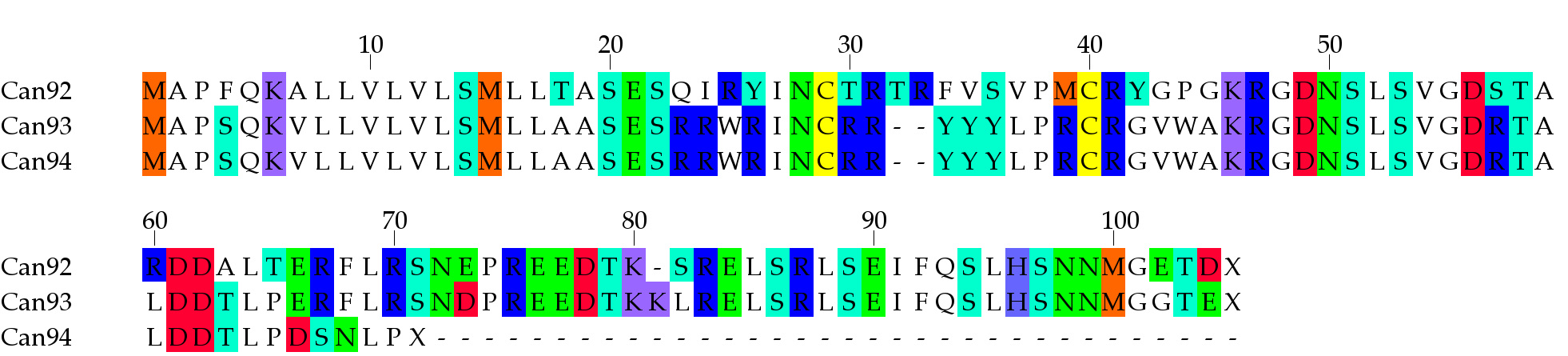


Figure S19. Sequence alignment of three conotoxin precursors of the Elevenin gene superfamily (Can92-Can94).

## Conopressin-Conophysins SUPERFAMILY


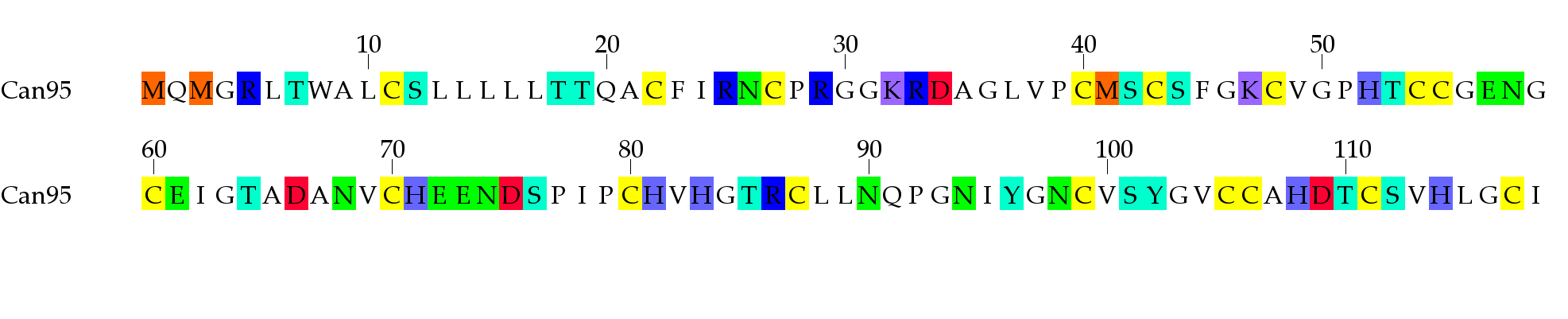


Figure S20. Sequence of a conotoxin precursor belonging to the Conopressin-conophysin gene superfamily (Can95).

## Conorfamide SUPERFAMILY


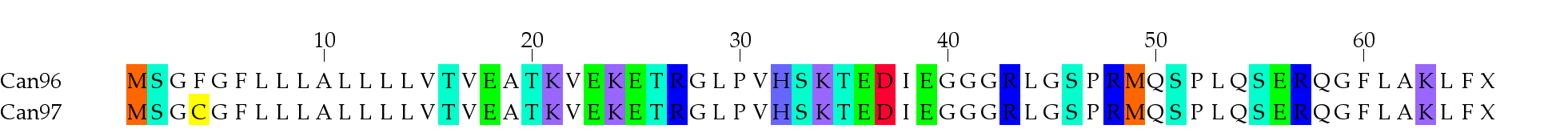


Figure S21. Sequence alignment of two conotoxin precursors of the Conorfamide gene superfamily.

## Neuropeptide prohormone-4 like


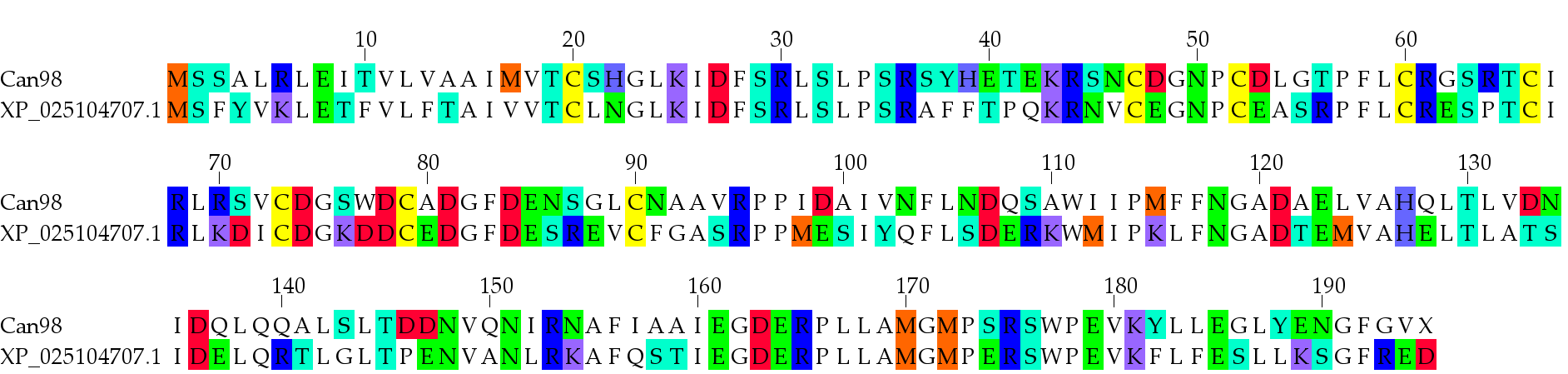


Figure S22. Sequence a conotoxin precursor (Can98) belonging to the neuropeptide prohormone 4 gene superfamily. Gene superfamily attribution was annotated by similarity with a neuropeptide prohormone previously identified in another gastropod, the golden apple snail *Pomacea* *canaliculate* (accession XM_025248922.1, Zhou et al., *Mitochondrial DNA Part A,* 2016).

# Proteomics

Results below represents the conotoxins that were additionnally validated through proteomics.

## E SUPERFAMILY


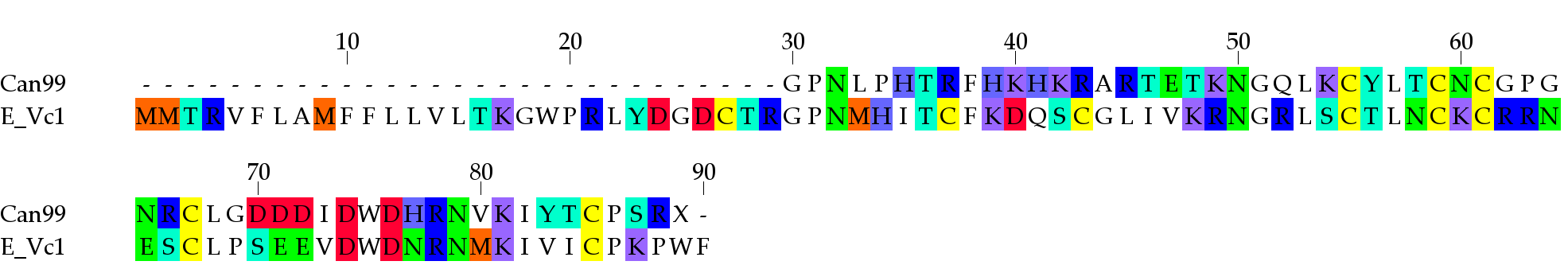


Figure S23. Sequence alignment of Can99, an E-conotoxin precursor identified in this study in the venom of *C. canonicus* with the E-conotoxin Vc1.1 from *C. victoriae* (Robinson et al., *PLOS ONE* 2014)*.*

## I1 SUPERFAMILY


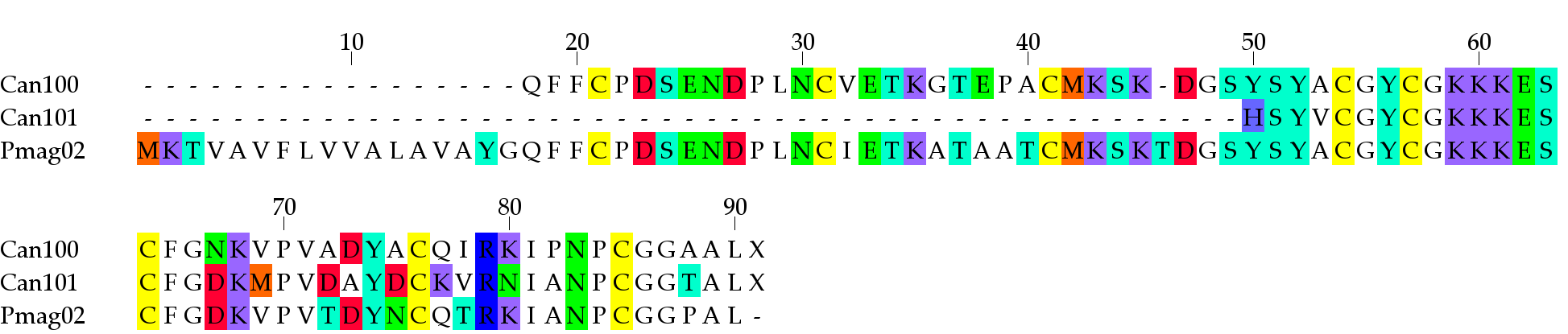


Figure S24. Sequence alignment of two I1-conotoxin precursors, identified in this study, in the venom of *C. canonicus* (Can100-Can101) with the I1-conotoxin Pmag02 from *C. ebraeus* (Pardos-Blas et al., *Mar. Drugs* 2022).

O1 SUPERFAMILY


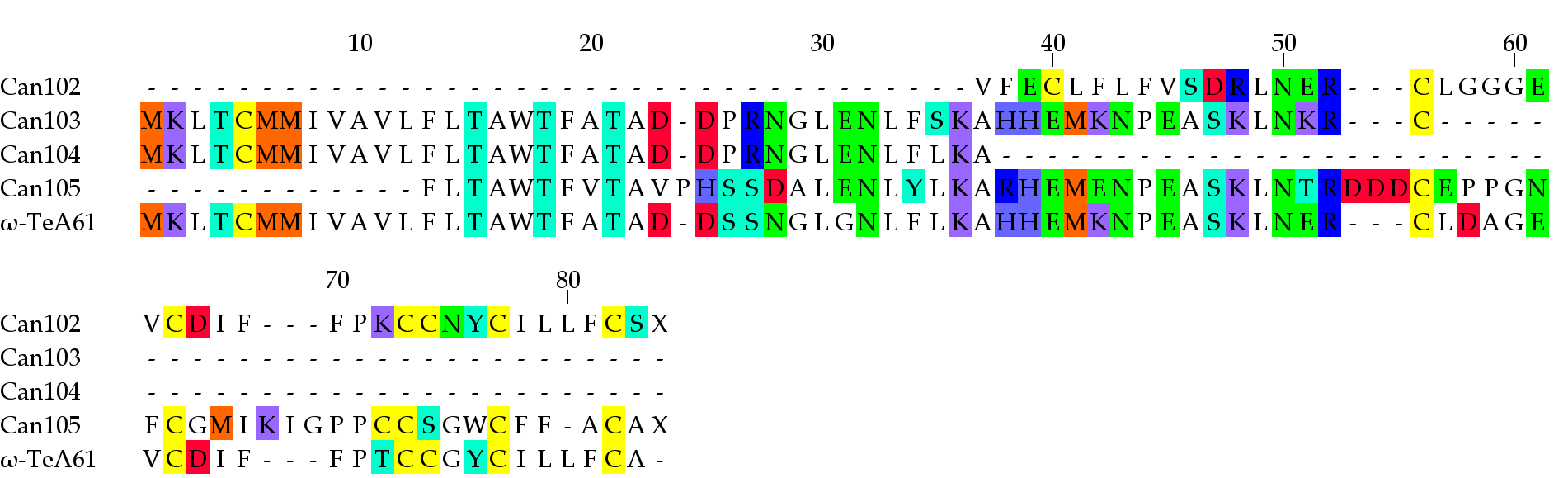


Figure S25. Sequence alignment of four O1-conotoxin precursors, from in this study, in the venom of *C. canonicus* (Can102-Can105) with the O1-conotoxin ω-TeA61 from *C. textile* (Luo et al., *J. Pept. Sci.* 2006)*.*

## O2 SUPERFAMILY


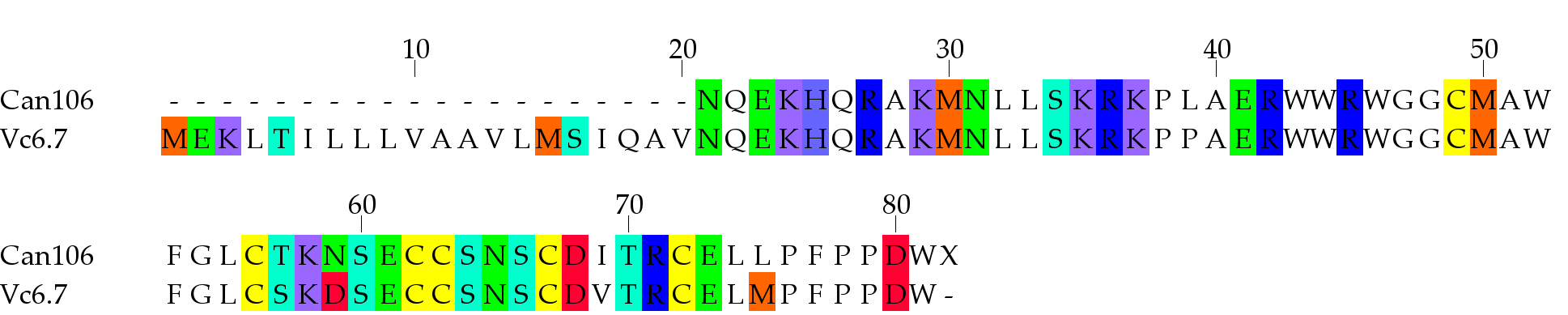


Figure S26. Sequence alignment of an O2-conotoxin precursor, from in this study, in the venom of *C. canonicus* (Can106) with the O2-conotoxin Vc6.7 from *C. victoriae* (Safavi-Hemani et al., *J. Proteome Res.* 2011)*.*

## T SUPERFAMILY


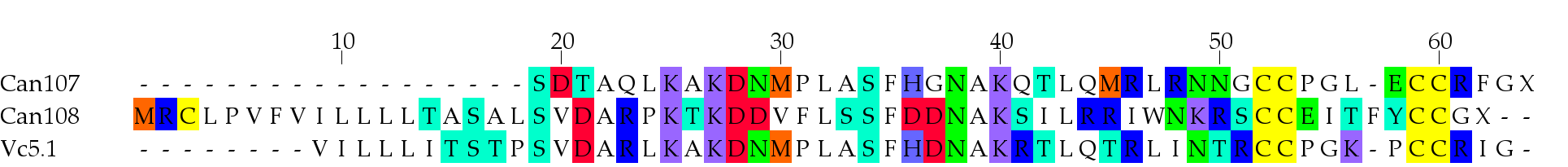


Figure S27. Sequence alignment of four T-conotoxin precursors, from in this study, in the venom of *C. canonicus* (Can107-Can108) with the T-conotoxin Vc5.1 from *C. victoriae* (Jakubowski et al., *J. Mass Spectrom.* 2004).
